# Supplementary material for: The interactome of intact mitochondria by cross-linking mass spectrometry provides evidence for coexisting respiratory supercomplexes
Source: Mol Cell Proteomics. 2017 Dec 8;17(2):216–32. doi: 10.1074/mcp.RA117.000470 (PMC5795388; doi:10.1074/mcp.RA117.000470)
Supplement: Supplemental Data [file supp_RA117.000470_133922_0_supp_23978_fzffwf.zip › spectra_annotation/mito_spectra_annotation/319-1-5-1-8-1.pdf]

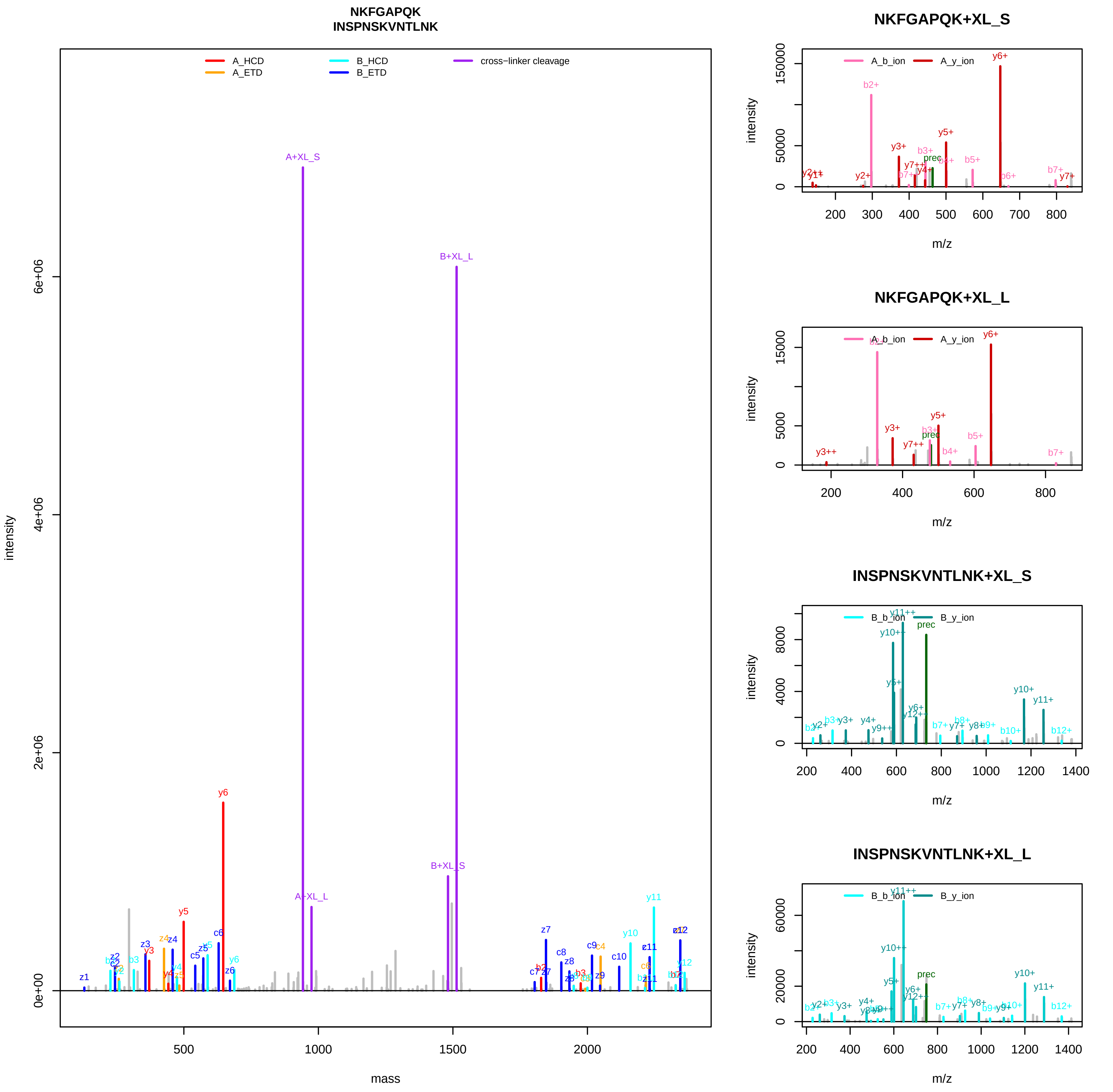

NKFGAPQK
INSPNSKVNTLNK
A_HCD
A_ETD
B_HCD
B_ETD
cross−linker cleavage
A+XL_S
B+XL_L
B+XL_S
A+XL_L
y6
y5
y11
y10
z12
intensity
0e+00
2e+06
4e+06
6e+06
mass
500
1000
1500
2000
NKFGAPQK+XL_S
NKFGAPQK+XL_L
INSPNSKVNTLNK+XL_S
INSPNSKVNTLNK+XL_L
A_b_ion
A_y_ion
B_b_ion
B_y_ion
y6+
b2+
y5+
y3+
prec
y11++
y10++
y10+
y11+
m/z
